# Dual attitudes toward doctors: the role of shared decision-making and empathy in frustrated medical treatment in a randomized trial

**DOI:** 10.3389/fpubh.2026.1888203

**Published:** 2026-07-29

**Authors:** Liang Ying, Yating Chen, Xinru Wang, Junting Huang, Jiani Yan, Jinyan Song, Qiang Zhou

**Affiliations:** 1 Wenzhou Medical University, Wenzhou, China; 2 Cixi Biomedical Research Institute, Wenzhou Medical University, Ningbo, China

**Keywords:** doctor-patient communication, doctor-patient relationship, dual attitude, empathy, shared decision making

## Abstract

**Objectives:**

Patient attitudes have a direct impact on treatment adherence, health status, and satisfaction. This study is to investigate whether increasing physician empathy and patient involvement can improve patient attitudes in the context of frustration.

**Methods:**

The study used a three-factor between-subjects design. 186 medical students initiated the situation through the recall imagination paradigm, watched the video of the simulated doctor-patient communication situation, made medical decisions in different ways, and measured explicit and implicit attitudes.

**Results:**

The results showed that implicit attitudes tended to be stable, while explicit attitudes were more variable, and the two were not significantly correlated, but empathic interpretation and shared decision-making significantly improved patients' explicit attitudes. The experimental group showed a more positive explicit attitude and higher satisfaction than the control group under the intervention of empathic interpretation and shared decision-making, especially in the frustration situation.

**Conclusion:**

Empathic explanation and shared decision-making have a positive effect on patients' negative explicit attitudes in frustrating medical treatment contexts. Improving physician empathy in doctor–patient communication and implementing shared decision-making may help enhance patients' communication experience, perceived trust, and evaluations of the doctor–patient interaction.

## 1 Introduction

Communication is the cornerstone of a good doctor–patient relationship (1). When doctors are good communicators, patient adherence doubles (2). However, patient frustration in healthcare settings is a common yet underappreciated phenomenon, often arising from long waiting times, poor communication, unmet expectations, or perceived lack of respect. Such frustration has been consistently associated with reduced satisfaction, diminished trust in healthcare providers, and poorer health outcomes (3). Although doctor–patient disputes represent a significant real-world challenge—with ineffective communication cited as a contributing factor in up to 70% of cases (4, 5)—the present study focuses on understanding the underlying psychological mechanisms, particularly how patient attitudes are shaped by communication processes. Therefore, effective doctor-patient communication is particularly important for establish a stable doctor–patient relationship and address the root causes of dissatisfaction (6). The most widely advocated mode of communication in medicine is patient-centered communication. The core of this mode is that doctors can fully understand and empathize with patients' needs and feelings. Physician empathy is an important factor in patient-centered communication (7).

Empathy refers to the ability to understand a patient's situation, views, and feelings; communicate this understanding to the patient; and act on this understanding in a beneficial therapeutic way. This definition provides a consistent framework that can guide both the teaching of empathy in medical schools and the assessment of such educational interventions (8). Research shows that a lack of empathy contributes to nearly 80% of doctor–patient disputes attributed to poor communication in medical interactions between medical staff and patients. Doctors with high levels of empathy can be aware of and deeply experience patients' emotions, better understand and support patients, and thus increase doctor–patient trust and improve a doctor–patient relationship (9, 10). Good doctor–patient communication can promote positive changes in patients' attitudes and prevent medical disputes (11, 12). Social norms, professional expectations, and the desire to maintain a good relationship with providers often lead patients to express positive attitudes toward doctors and healthcare. However, negative experiences or setbacks—such as perceived rudeness, feeling dismissed, or disappointing treatment outcomes—can create negative implicit or explicit attitudes that may not be openly disclosed, especially in Frustrated Medical Treatment. Understanding the basic attitudes of patients helps in conceptualizing patient-centered medical services, thereby improving the doctor–patient relationship through the design of medical education courses and medical activity systems (13). Studies have shown that implicit and explicit attitudes have different patterns of association with treatment decisions (14). Implicit attitudes can influence treatment decisions and patient health outcomes (15). Therefore, a comprehensive assessment of patients' attitudes toward healthcare professionals must consider both implicit and explicit attitudes, which are key to understanding patient satisfaction (16). In this context, shared decision-making (SDM) has been shown to be an effective method that can significantly change patients' attitudes by involving them in the decision-making process so that they can make more personalized treatment choices according to their own preferences and needs (17).

In recent years, the doctor–patient relationship has evolved from a paternalistic model to a collaborative SDM relationship. SDM is a collaborative process in which patients and physicians work together to combine patient values and preferences with clinical evidence to arrive at patient-centered decisions (18). In the SDM process, the first step is for doctors to present all possible treatment options to patients in detail, including their benefits and potential risks; next, patients need to express their wishes to doctors and share their views and concerns about different treatment options; finally, doctors and patients work together to make appropriate and rational choices regarding patients' medical problems. Studies have found that the use of SDM has a positive impact on patient involvement (19) doctor–patient relationships (20), and patient satisfaction (21–23).

In summary, previous studies have focused on the role of doctor–patient communication, empathy, attitude, and shared decision-making in reducing doctor–patient conflict and establishing a good doctor–patient relationship. Although these factors are essential for improving the doctor–patient relationship, many challenges remain in their practical application. Therefore, this study is based on patient-centered medical care and examines whether it can help improve patients' negative attitudes by enhancing doctor–patient communication, empathic interpretation, and patients' participation in medical decision-making in the context of frustrated medical treatment. Hypothesis 1 is as follows: frustrated medical treatment will lead participants to hold more negative explicit attitudes toward doctors and healthcare. Based on the importance of empathy in doctor–patient communication, we hypothesized that empathic explanation may help alleviate patients' negative attitudes. Therefore, Hypothesis 2 is as follows: Participants in the empathic explanation group will show more positive explicit attitudes than those in the non-empathic explanation group. In addition, since shared decision-making may affect participants' satisfaction and treatment adherence, this paper further explores the effect of shared decision-making on patients' attitudes. Hypothesis 3 is as follows: Participants in the shared decision-making group will show more positive explicit attitudes than those in the doctor's arbitrary decision-making group.

## 2 Methods

The study followed a 2 × 2 × 2 between-subjects design, which adopted a scenario-based experimental approach. Medical students were recruited to serve as simulated patients, enabling controlled manipulation of the key variables—empathy and decision-making—which represents a methodological advantage over real-world clinical settings. The dependent variables comprised patients‘ explicit attitudes, conversation satisfaction, medical decision-making satisfaction, and implicit attitudes. The aim was to explore the effects of patient involvement in the medical decision-making process and the presence or absence of empathic explanations in doctor-patient communication on patients' explicit and implicit attitudes in the context of frustrated medical treatment.

### 2.1 Participants

Second-year medical students from a medical university in China were recruited as participants through announcements in core medical courses. The inclusion criteria were as follows: second-year medical students who voluntarily agreed to participate, had normal or corrected-to-normal vision, were able to complete the computer-based experimental tasks, and had not participated in similar experiments before. The exclusion criteria were a self-reported history of mental disorders, visual or auditory impairments that could interfere with task performance, failure to complete the experiment, or an SC-IAT error rate ≥20%.

In the Chinese medical education system, students enter medical school directly after high school. By the end of their second year, they have acquired basic medical knowledge and clinical communication skills but have not yet engaged in independent clinical practice. This makes them particularly suitable for the present study, as they are able to adopt the perspectives of those with medical knowledge and clinical communication skills but not practicing physicians during the scenario-based simulation (24). However, medical students may differ from the general patient population because of their medical knowledge and possible pre-existing positive attitudes toward physicians. Therefore, these sample characteristics should be considered when interpreting the findings. Based on the experimental design of this study, G-Power 3.1 calculated the required sample size for the multivariate between-subjects analysis of variance used in this study to be 160 participants (α = 0.05, *f* = 0.3, *1-*β = 0.95). A total of 206 participants were recruited. The SC-IAT (Single Category Implicit Association Test), a reaction-time task used to measure implicit attitudes, was administered. Following previous SC-IAT scoring recommendations, 20 participants with an error rate ≥ 20% on the SC-IAT were excluded to ensure the validity of reaction-time-based D-scores (25). A total of 186 medical students were finally included in the analysis [age: *M* = 19.46 years, *SD* = 1.28 years; gender: 94 females (50.5%), 92 males (49.5%)]. The complete grouping of participants is shown in Table 1.

**Table 1:** Sample size of men and women within each condition and total sample size.

Medical situation communication decision-making			Female	Male	Sample size
Frustration	No empathic explanation	SDM	13	11	24
		Arbitrary	9	14	23
	Empathic explanation	SDM	13	9	22
		Arbitrary	10	13	23
Neutral	No empathic explanation	SDM	13	12	25
		Arbitrary	15	8	23
	Empathic explanation	SDM	12	13	25
		Arbitrary	9	12	21
Total(*N*)			94	92	186

### 2.2 Measures

#### 2.2.1 Induction of frustrated medical treatment

A recall/imagination paradigm was used to induce frustration using a medical event experienced by the subject without creating a story situation for the subject. The elicitation process was as follows: an 800ms fixation point appeared in the center of the computer screen, and then the participants were asked to recall a personal experience of frustrated medical treatment and then to write down the time, place, and process of the event, as well as the subject's psychological feelings, and to hand them over to the experimenter after completing the record.

#### 2.2.2 Doctor-patient communication

Drawing on real-life medical situations, a written script of doctor-patient communication was written in advance and divided into two scenarios: doctors with empathy and explanation and doctors without empathy and explanation, which were made into a video using video production software, and 10 college students who did not participate in this experiment rated the doctors' ability to empathize and explain on a scale of 1–7, and the difference in the ratings of the videos with and without empathy and explanation was significant (Appendix 1).

#### 2.2.3 Patient participation

Guideline for joint decision-making: After the examination, the doctor in the video gives two treatment options: A short treatment, good effect, but prone to adverse reactions; B long treatment, average effect, but less prone to adverse reactions. Please carefully consider and press (A/B) to choose the treatment option you think is better. Doctor's arbitrary decision-making guide: After the examination, the doctor in the video did not ask your wishes and directly chose the following treatment plan: a short course of treatment, effective but prone to adverse reactions. To understand, please press “space” to continue.

#### 2.2.4 Patient perceived doctor's service attitude scale

The questionnaire contained 23 questions, three of which were not applicable to the brief video consultation scenarios—namely, “The doctor makes regular rounds (attends outpatient visits).” “The doctor considers how to save costs for me” and “The doctor protects my privacy” were removed, resulting in a 20-item version used for the pre- and post-tests, and patient attitudes were assessed on a scale of 1-7 from “strongly disagree” to “strongly agree.” The split-half reliability and Cronbach's alpha of the scale are above 0.8, which can be considered good reliability (26). The questionnaire posttest added two satisfaction score items to test the effectiveness of doctor-patient communication and patient participation in decision-making (Appendix 2).

#### 2.2.5 SC–IAT

Patients' implicit attitudes were measured using the SC-IAT, and the experiment was prepared by Psychopy v2021. 2.3. Psychology graduate students who did not participate in this study followed standard procedures for selecting concept and attribute words. Concept words referring to physicians included the following seven terms: doctor, physician, medical staff, healthcare worker, clinician, general practitioner, and specialist. Twenty positive attribute words (e.g., sincere, kind, successful, noble, competent, etc.) and twenty negative attribute words (e.g., selfish, cold-blooded, hateful, arrogant, etc.) were used. The entire experiment was divided into two task types with four phases, two practice phases and two formal experimental phases, where participants were given feedback on the accuracy of their responses (27). To balance the number of keystrokes, the ratio of keystrokes was positive words: Conceptual Words: Negative Words = 7:7:10. The IAT scores refer to the method of calculating SC-IAT scores as proposed by Greenwald et al., and the D-scores were used to indicate the implicit effect (Appendix 3).

### 2.3 Design

**Figure 1:**
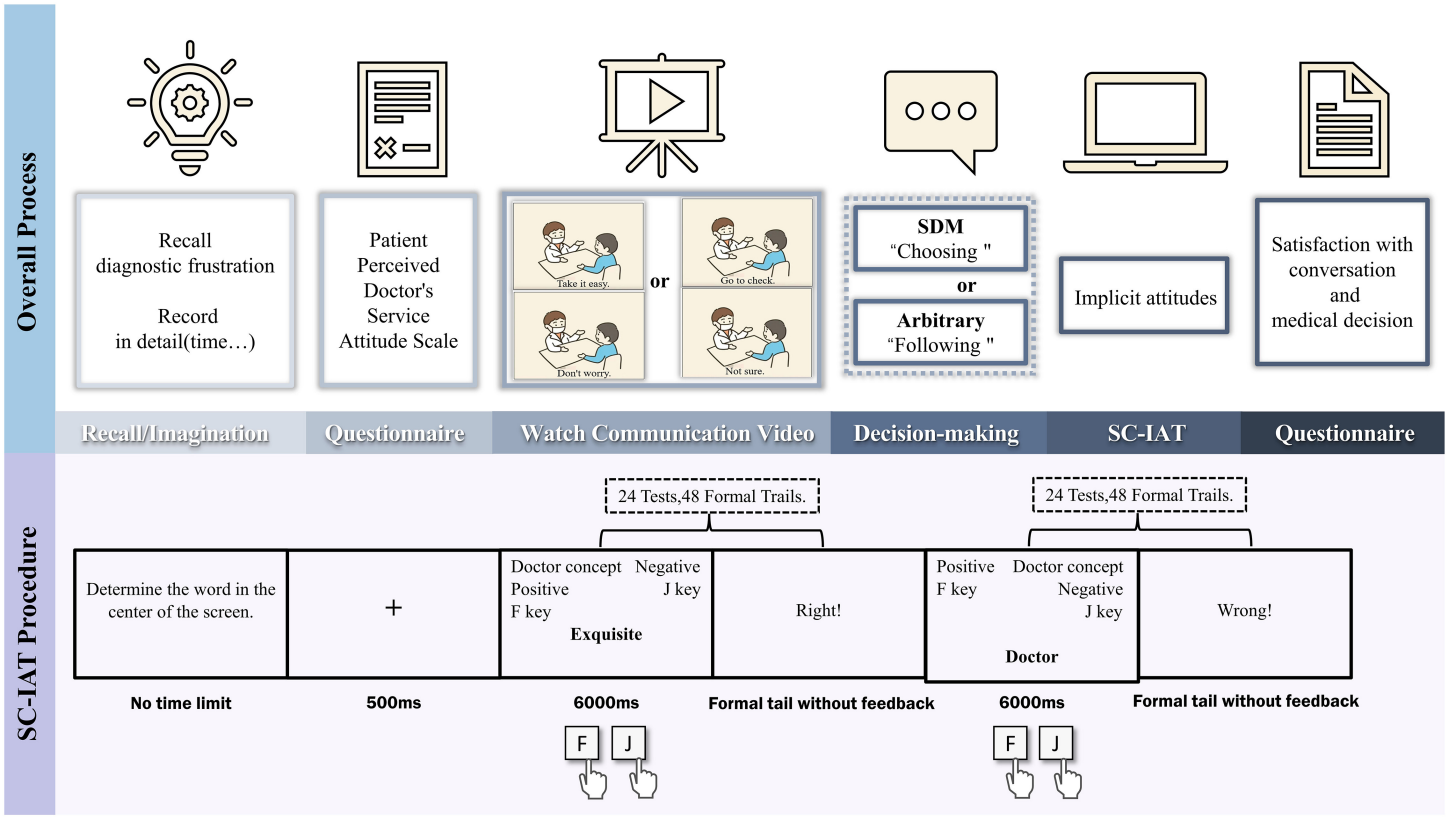
Flow diagram of study design.

The experimental procedure referred to the patient emotion cue task paradigm (28) and was programmed using PsychoPy 3.0 (Open Science Tools Ltd., Nottingham, England). The stimuli were presented on a 23-inch display with a black background [RGB (0, 0, 0)], a resolution of 1024 × 768, and a refresh rate of 60 Hz. First, participants were prompted by the guidance to enter the context initiation stage, and the frustrated or neutral diagnosis and treatment events were recalled and recorded in groups to ensure the effectiveness of the experimental situation. Subsequently, an explicit questionnaire pre-test was conducted to verify the effect of context initiation. This pre-test was used as an indirect quantitative manipulation check to examine whether the recall/imagination task successfully induced differences in participants' attitudes between the frustration and neutral conditions. Then, the participants watched the doctor-patient communication video and participated in the medical decision-making task based on the video content to experience the different situations of doctor-patient shared decision-making and doctor-arbitrary decision-making. After the decision-making, the explicit questionnaire post-test was conducted to evaluate the participants' satisfaction with the conversation and medical decision-making. Finally, the one-class implicit association test (SC–IAT) was used to assess the participants' positive or negative attitudes toward doctor-related vocabulary, including the practice and test phases of compatible and incompatible tasks, aiming to reveal the changes in participants‘ attitudes (Figure 1). A single-blind design was used, in which the experimenter knew the procedure, while participants did not. We drafted this manuscript in accordance with the CONSORT reporting guidelines (29), please refer to the Appendix 4 for the checklist.

### 2.4 Statistical analysis

All statistical analyses were performed using SPSS 23.0. In order to test the effect between conditions, an analysis of variance between subjects was performed, and the significance level was set at *P* < 0.05. For simple-effects analyses following significant interaction effects, the Bonferroni correction was applied to adjust for multiple comparisons. Descriptive statistics were reported with 95% confidence intervals.

## 3 Results

### 3.1 Manipulation check for medical situation induction

To examine whether the paradigm effectively induced different medical situations, we compared the explicit attitude pre-test scores between the frustration and neutral conditions. The main effect of medical situation was significant, *F*_(1, 184)_ = 4.265, *p* < 0.05, $\eta_{p}^{2}$ = 0.023. Participants in the frustration condition reported lower explicit attitude scores [*M* ±*SD* = 5.478 ± 1.132, 95% *CI* = (5.243, 5.713)] than those in the neutral condition [*M* ± *SD* = 5.821 ± 1.130, 95%*CI* = (5.589, 6.052)]. These results indicate that the paradigm induced different explicit attitudinal responses between the frustration and neutral conditions.

### 3.2 Patient explicit attitude

**Figure 2:**
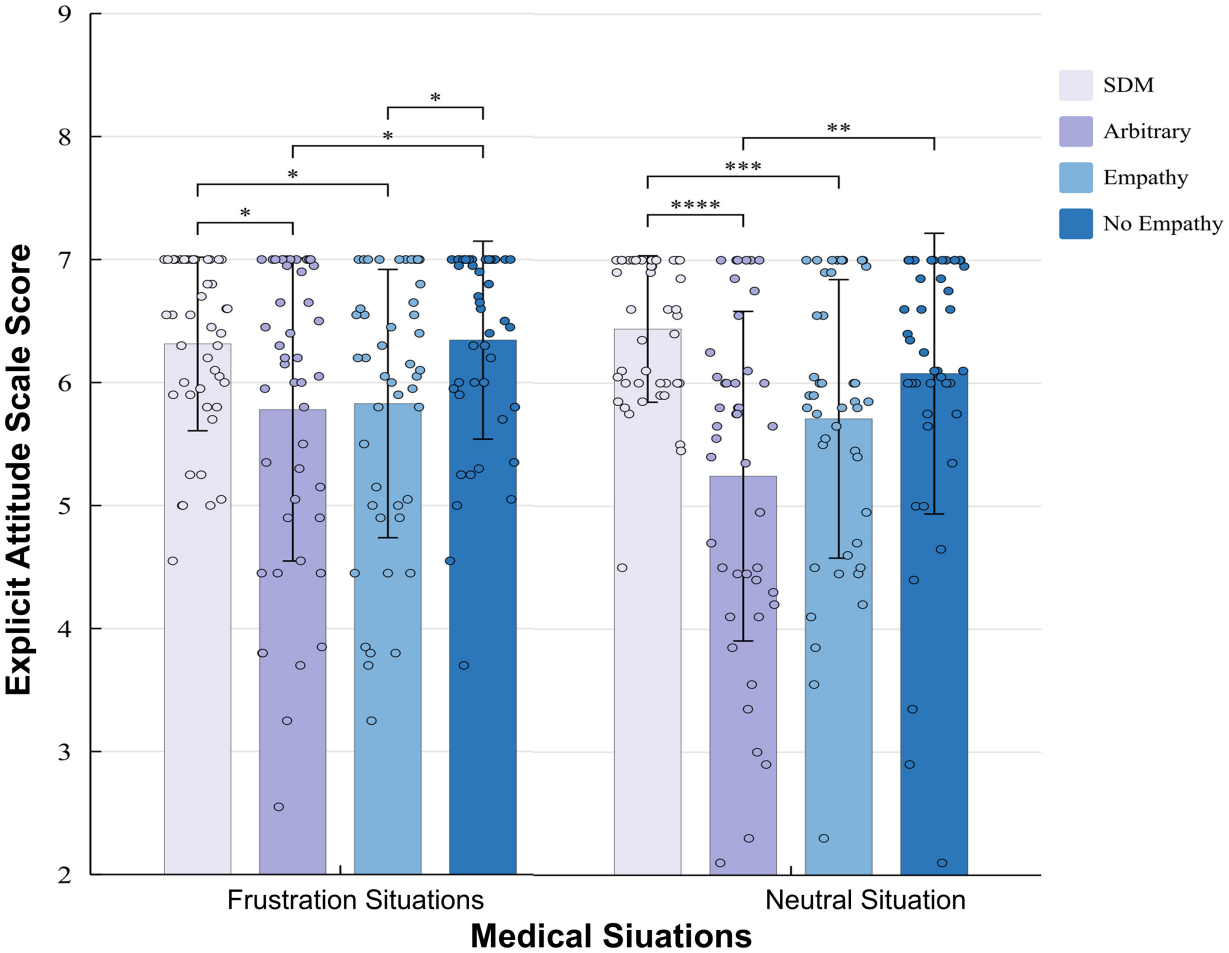
The effect of shared decision-making and empathy on patient attitudes in different contexts.

The main effect of the medical situation was significant [*F* (1, 178) = 4.22, *p* < 0.05, $\eta_{p}^{2}$ = 0.02], indicating that participants' explicit attitudes differed significantly between the frustration and neutral conditions. The main effect of doctor-patient communication style was significant [*F*_(1, 178)_ = 10.03, *p* < 0.01, $\eta_{p}^{2}$ = 0.05], suggesting that empathic explanation was associated with more positive explicit evaluations of doctors. In addition, the main effect of decision-making style was significant [*F*_(1, 178)_ = 19.66, *p* < 0.001, $\eta_{p}^{2}$ = 0.10], showing that decision-making style explained a relatively larger proportion of variance in explicit attitudes than the other two main factors. The interaction of the three was significant [*F*_(1, 178)_ = 7.16, *p* < 0.01] (Figure 2), indicating that the influence of empathic explanation and decision-making style differed across frustrating and neutral medical situations. To further examine this significant interaction, simple-effects analyses were performed with Bonferroni correction for multiple comparisons. The corrected results showed that, in frustrating situations and arbitrary decision-making, the post-test score of the explicit questionnaire with an empathic explanation was significantly higher than that without an empathic explanation [*F*_(1, 178)_ = 17.83, *p* < 0.001, $\eta_{p}^{2}$ = 0.09]. When there was a frustrating situation and the video-depicted doctors had no empathic explanation, the post-test score of the explicit questionnaire under common decision-making was significantly higher than that of doctors‘ arbitrary decision-making [*F*_(1, 178)_ = 13.86, *p* = 0.001, $\eta_{p}^{2}$ = 0.07]. In a neutral situation and without empathic explanation, the post-test score of the explicit questionnaire under shared decision-making was significantly higher than that of arbitrary decision-making [*F*_(1, 178)_ = 7.48, *p* = 0.027, $\eta_{p}^{2}$ = 0.04]. When doctors had empathic explanations in neutral situations, the explicit questionnaire post-test score under common decision-making was significantly greater than that of arbitrary decision-making [*F*_(1, 178)_ = 11.46, *p* = 0.004, $\eta_{p}^{2}$ = 0.06] (Table 2).

**Table 2:** Between-subjects ANOVA for the posttest of the exogenous questionnaire.

Source	*SS*	*MS*	*F(df)*	*η^2^*
Frustration	5.109	5.109	4.222(1,178) ^**^	0.023
Empathy	12.132	12.132	10.026(1,178) ^**^	0.053
Decision-making	23.794	23.794	19.663(1,178) ^***^	0.099
Frustration^*^Empathy	0.564	0.564	0.466(1,178)	0.003
Frustration^*^Decision-making	3.359	3.359	2.776(1,178)	0.015
Empathy^*^Decision-making	4.613	4.613	3.812(1,178)	0.021
Frustration^*^Empathy^*^Decision-making	8.658	8.658	7.155(1,178) ^**^	0.039

SS, sum of squares of class III; MS, mean square; η^2^, effect size; ^*^p < 0.05; ^**^p < 0.01.

### 3.3 Satisfaction with conversation

The main effect of simulated doctor-patient communication style was significant [*F*_(1, 178)_ = 10.51, *p* < 0.001, $\eta_{p}^{2}$ = 0.06], indicating that empathic explanation significantly improved participants' satisfaction with the conversation. The interaction between communication style and medical decision-making was also significant [*F*_(1, 178)_ = 10.51, *p* < 0.05], suggesting that the effect of empathic explanation varied according to the decision-making context. To further examine this significant interaction, simple-effects analyses were performed with Bonferroni correction for multiple comparisons. The corrected result showed that, under arbitrary decision-making, conversation satisfaction without empathic explanation was significantly lower than that with empathic explanation [*F*_(1, 182)_ = 15.38, *p* < 0.001, $\eta_{p}^{2}$ = 0.08]. In frustrating situations and when making arbitrary decisions, participants' satisfaction with conversations with empathic explanations was significantly greater than that without empathic explanations [*F*_(1, 178)_ = 22.21, *p* < 0.001, $\eta_{p}^{2}$ = 0.11]. The interaction of medical situation, communication style, and medical decision-making was significant [*F*_(1, 178)_ = 8.44, *p* < 0.01].

### 3.4 Satisfaction with decision making

The main effect of medical decision-making was significant [*F*_(1, 178)_ = 60.99, *p* < 0.001, $\eta_{p}^{2}$ = 0.26], and the satisfaction of medical decision-making of shared decision-making was significantly greater than that of arbitrary decision-making. The relatively large $\eta_{p}^{2}$ value indicates that decision-making style explained a substantial proportion of the variance in decision-making satisfaction. The interaction between medical situation and medical decision-making was significant [*F*_(1, 178)_ = 12.55, *p* < 0.001]. To further examine this significant interaction, simple-effects analyses were performed with Bonferroni correction for multiple comparisons. The corrected results showed that satisfaction with medical decision-making under shared decision-making was significantly greater than that under arbitrary decision-making in both frustrating situations [*F*_(1, 182)_ = 7.96, *p* = 0.011, $\eta_{p}^{2}$ = 0.04] and neutral situations [*F*_(1, 182)_ = 55.87, *p* < 0.001, $\eta_{p}^{2}$ = 0.24]. This suggests that shared decision-making improved satisfaction across different medical situations, with a stronger effect observed in the neutral condition. There was a significant interaction between empathic interpretation and medical decision-making [*F*_(1, 178)_ = 7.55, *p* < 0.01]. Without empathic interpretation, the satisfaction of medical decision-making of SDM was significantly greater than that of arbitrary decision-making [*F*_(1, 182)_ = 48.14, *p* < 0.001, $\eta_{p}^{2}$ = 0.21], with empathic explanation, the satisfaction of medical decision-making of SDM was significantly greater than that of arbitrary decision-making [*F*_(1, 182)_ = 9.80, *p* = 0.004, $\eta_{p}^{2}$ = 0.05]. The interaction of medical situation, communication style, and medical decision-making was significant [*F*_(1, 178)_ = 15.14, *p* < 0.001].

### 3.5 Patients' implicit attitude

When the implicit mean is 0, the implicit attitude of the IAT on behalf of the individuals is neutral, neither positive nor negative. Therefore, the implicit mean D-value and 0 were tested by a sample t-test (Table 3): the D–value of each group was greater than 0, indicating that the implicit attitude of the eight groups of participants was generally positive.

**Table 3:** D-value and one-sample t-test for implicit attitudes toward medicine in SC-IAT.

Medical situation communication decision-making			*D* Value	*SD*	*t*	*P* Value
Frustration	No empathic explanation	SDM	0.050	0.510	0.484	0.633
		Arbitrary	0.353	0.441	3.919	< 0.001^***^
	Empathic explanation	SDM	0.230	0.511	2.680	0.014^*^
		Arbitrary	0.259	0.426	2.915	0.008^**^
Neutral	No empathic explanation	SDM	0.353	0.339	5.198	< 0.001^***^
		Arbitrary	0.289	0.432	3.204	0.004^**^
	Empathic explanation	SDM	0.310	0.427	3.627	0.001^**^
		Arbitrary	0.338	0.562	2.759	0.012^*^

SD, Standard deviation; ^*^p < 0.05; ^**^p < 0.01; ^***^p < 0.001.

The D-value in neutral situation (*M* ± *SD* = 0.32 ± 0.44) was greater than that in frustration situation (*M* ± *SD* = 0.24 ± 0.48), indicating that frustration situation will reduce positive attitude, which is consistent with the pretest results of the explicit questionnaire. There was no significant effect of doctor–patient communication style and whether patients participated in decision-making [(*F*_(1, 178)_ = 1.36, *p* = 0.246, $\eta_{p}^{2}$=0.01; *F*_(1, 178)_ = 0.23, *p* = 0.629, $\eta_{p}^{2}$= 0.001; *F*_(1, 178)_ = 0.91, *p* = 0.340, $\eta_{p}^{2}$= 0.01)], there was no significant correlation between patients' explicit attitude and implicit attitude (*r* = −0.02, *p* = 0.782). The repeated measures ANOVA of mean reaction time found that: 1) the main effect of group was not significant [*F*_(7, 178)_ = 1.19, *p* = 0.086, $\eta_{p}^{2}$= 0.05]; 2) the main effect of task type was significant [*F*_(1, 178)_ = 45.93, *p* < 0.001, $\eta_{p}^{2}$= 0.02]; 3) the interaction between group and task type was significant [*F*_(7, 178)_ = 2.28, *p* = 0.030] (Table 4).

**Table 4:** Average response time for compatible and incompatible tasks in SC-IAT.

Medical situation communication decision-making			Compatibility experiment (ms)	Incompatibility experiment (ms)	Difference
Frustration	No empathic explanation	SDM	772.851	774.342	1.490
		Arbitrary	667.819	727.604	59.785
	Empathic explanation	SDM	669.214	723.885	54.671
		Arbitrary	644.566	694.992	50.425
Neutral	No empathic explanation	SDM	677.939	784.088	106.149
		Arbitrary	734.441	775.899	41.458
	Empathic explanation	SDM	676.878	738.961	62.083
		Arbitrary	714.448	776.472	62.024

Difference, Incompatible response time - compatible response time.

## 4 Discussion

### 4.1 Main findings

This study aimed to investigate the influence of patient involvement and doctor–patient communication in medical decision-making on patient attitudes under conditions of frustration with medical treatment. We found that patients' explicit attitudes were significantly influenced by external situations, whereas their implicit attitudes were relatively stable, which is consistent with previous research (30). Although participants only had to recall their medical experiences within two years, frustrated medical treatment still caused negative explicit attitudes, suggesting that patients' negative attitudes toward medical staff may be difficult to eliminate in the short term. From the perspective of behavioral medicine, frustrating medical experiences may activate negative emotional appraisal, reduce perceived control, and weaken patients' expectations of being respected during medical encounters. These psychological responses may then be reflected in more negative explicit evaluations of doctors. However, the relationship between explicit and implicit attitudes was not statistically significant. The research findings are consistent with the dual attitude theory, that is, explicit and implicit attitudes are completely different psychological concepts that can exist independently of each other and may have different effects in specific situations (31, 32). This dissociation may occur because explicit attitudes are more sensitive to recent experiences, conscious appraisal, and situational information, whereas implicit attitudes are based on relatively automatic associations accumulated over time and may therefore be less susceptible to short-term experimental manipulations. Therefore, when analyzing patients' attitudes, we should not only focus on one dimension of attitudes but should also comprehensively consider both explicit and implicit attitudes to obtain a true and credible understanding of patients' attitudes. Fortunately, the study showed that the D-value of patients' implicit attitudes was generally greater than zero, indicating that patients' implicit attitudes were positive and stable.

In addition, we found that empathic explanation significantly influenced patients' attitudes and satisfaction, which is consistent with previous research (10). Specifically, explicit questionnaire scores—indicating more positive attitudes toward the doctor—were higher in the empathic explanation condition than in the non-empathic explanation condition, and conversation satisfaction in the former was significantly higher than in the latter. This finding may be explained by the role of empathy in patient-centered communication. When doctors provide empathic explanations, patients may perceive that their emotions and concerns are recognized rather than ignored. Such communication can reduce uncertainty, relieve negative emotions, and strengthen the patient's perception of respect and support. In frustrating medical situations, this effect may be particularly important because empathic explanation can buffer negative emotional responses associated with prior frustrating experiences. Consistent with previous studies, physician empathy can improve patient satisfaction and establish a harmonious doctor-patient relationship (33). Therefore, we believe that strengthening the training of medical students in communication skills and empathy abilities is essential in medical education. As to why the empathic explanation did not significantly affect implicit attitudes, a ceiling effect may have occurred; that is, participants had already shown a very positive implicit attitude before the test (34).

We also found that, compared to participants who made arbitrary decisions, those who made shared decisions had significantly higher satisfaction with medical decisions, which is consistent with previous findings (35). SDM is based on reliable clinical medical evidence, combined with patients' values and decision orientation, and combines the professional knowledge and technology of doctors with the wishes and economic capabilities of patients. Therefore, shared decision-making has the “highest value for money,” which can help both doctors and patients make the best medical decisions (36). From a clinical communication perspective, the positive effect of SDM may result from increased patient autonomy and perceived control. When patients are invited to participate in treatment decisions, they are more likely to feel that their preferences, risks, and personal circumstances have been considered. This process may improve the perceived fairness of medical decisions and reduce the sense of passivity caused by doctor-dominated decision-making. Furthermore, as patients become more aware of health and self-care, their desire to participate in medical decision-making increases, and they can increasingly express their willingness to participate in medical decision-making rationally (37).

In summary, the results show that empathic explanation and SDM play crucial roles in shaping patients' attitudes and reveal the variability of explicit attitudes and stability of implicit attitudes. These findings suggest that empathy and shared decision-making are important components of patient-centered communication and may help medical students better understand patients' psychological responses in frustrating medical treatment contexts.

### 4.2 Limitations and further steps

There are still deficiencies in this study. First, all participants were second-year medical students acting as simulated patients, which may limit the generalizability of the findings. Their medical knowledge and prior attitudes toward doctors may differ from those of ordinary patients. Future studies should include real patients or community samples to further verify the present findings. Second, this study is a cross-sectional study between subjects, and there are individual differences. Therefore, follow-up research can be done within subjects. After the participants have negative attitudes, an intervention can be carried out to further study the participants‘ attitude change and its influencing factors. Third, the simulated medical scenarios and the induced frustration may not fully capture the complexity and emotional intensity of real-life frustrated medical treatment, which could limit the generalizability of the findings to actual clinical settings. Future studies should conduct real clinical experiments to further examine whether empathic explanation and shared decision-making influence patients' attitudes, satisfaction, and doctor–patient interaction outcomes in actual medical encounters. Finally, in terms of research results, this study has concluded that explicit attitude and implicit attitude are separated. Future research can add EEG or eye movement equipment to reveal the different neural mechanisms of explicit attitude and implicit attitude.

## 5 Conclusion

This study identified several key factors that influence patients' attitudes and analyzed the relationship between explicit and implicit attitudes. The results show that patients' attitudes tend to be negative in the context of frustrated medical treatment; however, negative attitudes can be significantly improved by empathic explanation between doctors and patients and a shared decision-making process in medical decision-making. Medical students can improve their empathy toward patients and learn to communicate effectively as future doctors. These findings highlight the importance of incorporating empathy and shared decision-making into medical communication education to cultivate patient-centered communication skills and more positive doctor–patient interaction experiences.

## Data Availability

The datasets presented in this study can be found in online repositories. The names of the repository/repositories and accession number(s) can be found below: https://osf.io/9s3wc?view_only=8faa5a99d903438882f6cd604af550d3.
