# Supplementary material for: Dual attitudes toward doctors: the role of shared decision-making and empathy in frustrated medical treatment in a randomized trial
Source: Front Public Health. 2026 Jul 29;14:1888203. doi: 10.3389/fpubh.2026.1888203 (PMC13461487; doi:10.3389/fpubh.2026.1888203)
Supplement: Supplementary file 1 [file Data_Sheet_1.pdf]

Appendix 1. Simulated scripts for doctor–patient interactions.

Empathic Explanation	Doctor: Hello, what's wrong?<br>Patient: Hello doctor, since I recovered from COVID that my heart is beating very fast, what's wrong with me?<br>Doctor：Okay, don't be nervous, can you tell me more about the specific symptoms?<br>Patient: After I recovered from COVID, I often feel panic and palpitations, my heart beats very fast, and I can't catch my breath, and it gets worse when I move around. I've asked people around me about this, and I'm worried that I might have myocarditis.<br>Doctor: OK, I understand, I can understand your concern, but in fact, after the new crown many reasons can cause the heart to beat faster, such as taking some drugs to relieve the symptoms of the new crown and so on.<br>Patient: So doctor is this serious in my case? Do I need any more tests?<br>Doctor: Many people do not need special treatment for a rapid heart rate, let alone medication to lower it. Generally speaking, this symptom will improve with the natural course of the disease, that is to say, when the body recovers for a few more days, the heart rate will gradually return to normal. Of course, in order to make an accurate judgement, it is recommended to have a blood test and an ECG, after which a specific judgement can be made.<br>Patient: OK, thank you doctor, I will go and have blood and tests done now.
No Empathic Explanation	Doctor: Hello, what's wrong?<br>Patient: Hello doctor, since I recovered from COVID that my heart is beating very fast, what's wrong with me?<br>Doctor: Any specific symptoms?<br>Patient: After I recovered from COVID, I often feel panic and palpitations, my heart beats very fast, and I can't catch my breath, and it gets worse when I move around. I've asked people around me about this, and I'm worried that I might have myocarditis.<br>Doctor：You can't be sure whether it is myocarditis, you need to do further examination.<br>Patient: So doctor is this serious in my case? Do I need any more tests?<br>Doctor: Go and take blood tests, and then do an electrocardiogram, and then make a specific judgement.<br>Patient: OK, thank you doctor, I will go to draw blood and do tests now.
