# Supplementary material for: Dual attitudes toward doctors: the role of shared decision-making and empathy in frustrated medical treatment in a randomized trial
Source: Front Public Health. 2026 Jul 29;14:1888203. doi: 10.3389/fpubh.2026.1888203 (PMC13461487; doi:10.3389/fpubh.2026.1888203)
Supplement: Supplementary file 2 [file Data_Sheet_2.pdf]

**Appendix 2.** Patient Perceived Physician Attitude Scale.

| Indicator | Measurement Statement |
|---|---|
| 1 | Doctors give me some comfort |
| 2 | The doctor pays attention to my feelings |
| 3 | The doctor made me feel relaxed |
| 4 | My doctor gives me confidence |
| 5 | My doctor is willing to help me |
| 6 | I feel that my doctor is trustworthy |
| 7 | My doctor understands how I feel |
| 8 | The doctor communicates with me as an equal |
| 9 | The doctor cares about the patient |
| 10 | The doctor is polite |
| 11 | The doctor answered my questions in detail |
| 12 | The doctor told me everything I wanted to know |
| 13 | The doctor answered my questions promptly |
| 14 | The doctor listened to me carefully |
| 15 | I understood what the doctor said |
| 16 | The doctor was patient with me |
| 17 | The doctor gave me enough time to communicate |
| 18 | I felt that the doctor did not prescribe tests indiscriminately |
| 19 | The doctor did what he promised to do in a timely manner |
| 20 | The doctor was serious when examining me |
| 21 | I was satisfied with the conversation I had with my doctor |
| 22 | I am satisfied with the medical decision I just made |
