# Supplementary material for: Dual attitudes toward doctors: the role of shared decision-making and empathy in frustrated medical treatment in a randomized trial
Source: Front Public Health. 2026 Jul 29;14:1888203. doi: 10.3389/fpubh.2026.1888203 (PMC13461487; doi:10.3389/fpubh.2026.1888203)
Supplement: Supplementary file 3 [file Data_Sheet_3.pdf]

Appendix 3. Implicit Attitude Measurement.

Type	Measurement vocabulary
Conceptual	Doctor, physician, healthcare worker, medical worker, doctor, general practitioner, specialist
Attribute	Positive attribute words: sincere, friendly, successful, noble, competent, likeable, excellent, enthusiastic, glory, dutiful, great, self-confident, outstanding, brave, upwardly mobile, sublime, diligent, kind, wise, friendly<br>Negative Attribute Words: selfish, cold-blooded, hateful, condescending, defeatist, loathsome, detestable, tiresome, obnoxious, fearful, greedy, corrupt, dark, shameful, mediocre, incompetent, decadent, atrocious, despicable, horrible

Step	Experimental Procedure
1	Compatibility task practice phase: Participants were asked to judge the words that appeared in the centre of the screen and to press the 'F' key if they were positive descriptions or positive attitudes and related to doctors; if they were negative descriptions or negative attitudes, they were asked to press the 'J' key. key' if the word was a negative description or negative attitude. There were 24 trials in the practice phase.
2	Compatible Tasks Test Phase: The key press requirements were the same as in Phase 1, and the quiz phase consisted of 48 trials.
3	Incompatibility task practice phase: Participants were asked to judge the words that appeared in the centre of the screen, and if they were positive descriptions or positive attitudes, they were required to press the 'F' key; if they were negative descriptions or negative attitudes and words related to doctors, they were required to press the 'J' key. 'key if the word was a negative description or attitude and related to doctors. There were 24 trials in the practice phase.
4	Incompatible Tasks Test Phase: The key press requirements were the same as in Phase 3, and the test phase consisted of 48 trials.

Step	Score Calculation Method
1	Remove data from experiments with an error rate greater than 20 per cent
2	Calculating the mean reaction time and standard deviation of correct responses for compatible and incompatible tasks for each participant
3	Modify the extremes and errors in each participant's data (replacing them with the mean reaction time of the correct response in the block to which they belonged plus 400ms).
4	Calculate the mean of all responses for the compatible and incompatible tasks for each participant
5	Calculate the difference between the mean reaction times for the compatible and incompatible tasks and divide by the standard deviation of reaction times for all correct responses (excluding the original incorrect response) to obtain a D-score, which represents the implicit effect.
6	The resulting data were subjected to descriptive statistics and tests of variance
