# Supplementary material for: Dual attitudes toward doctors: the role of shared decision-making and empathy in frustrated medical treatment in a randomized trial
Source: Front Public Health. 2026 Jul 29;14:1888203. doi: 10.3389/fpubh.2026.1888203 (PMC13461487; doi:10.3389/fpubh.2026.1888203)
Supplement: Supplementary file 4 [file Data_Sheet_4.pdf]

The CONSORT reporting checklist

For checking that reports of randomised trials can be understood and used by everyone

	Item Description	Location (or reason for not reporting)
**Title and Abstract**		
1a. Title	Identification as a randomised trial.	Line 3
1b. Structured Abstract	Structured summary of the trial design, methods, results, and conclusions.	Line 6-22
**Open Science**		
2. Trial Registration	Name of trial registry, identifying number (with URL) and date of registration.	Not pre-registered. This study is a non-clinical behavioral experiment; prospective trial registration is not applicable per ICMJE guidelines.
Protocol and statistical analysis plan	Where the trial protocol and statistical analysis plan can be accessed.	Paragraphs 11
4. Data sharing	Where and how the individual de-identified participant data (including data dictionary), statistical code and any other materials can be accessed.	Line 334
5. Funding and Conflicts of Interest		
5a. Funding	Sources of funding and other support (eg, supply of drugs), and role of funders in the design, conduct, analysis, and reporting of the trial.	Line 338
5b. Conflicts of interest	Financial and other conflicts of interest of the manuscript authors.	Line 345
**Introduction**		
6. Background and rationale	Scientific background and rationale.	Paragraphs 1
7. Objectives	Specific objectives related to benefits and harms.	Paragraphs 4
**Methods**		
8. Patient and public involvement	Details of patient or public involvement in the design, conduct and reporting of the trial.	Paragraphs 5

9. Trial Design	Description of trial design including type of trial (eg, parallel group, crossover), allocation ratio, and framework (eg, superiority, equivalence, non-inferiority, exploratory).	Paragraphs 11
10. Changes to trial protocol	Important changes to the trial after it commenced including any outcomes or analyses that were not pre-specified, with reason.	Not applicable; there were no changes to the trial protocol.
11. Trial Setting	Settings (eg, community, hospital) and locations (eg, countries, sites) where the trial was conducted.	Paragraphs 6
12. Eligibility Criteria		
12a. Participants	Eligibility criteria for participants.	Paragraphs 6
12b. Other	If applicable, eligibility criteria for sites and for individuals delivering the interventions (eg, surgeons, physiotherapists).	Not applicable
13. Intervention and comparator	Intervention and comparator with sufficient details to allow replication. If relevant, where additional materials describing the intervention and comparator (eg, intervention manual) can be accessed.	Paragraphs 7-10
14. Outcomes	Prespecified primary and secondary outcomes, including the specific measurement variable (eg, systolic blood pressure), analysis metric (eg, change from baseline, final value, time to event), method of aggregation (eg, median, proportion), and time point for each outcome.	Paragraphs 7-10
15. Harms	How harms were defined and assessed (eg, systematically, non-systematically).	Not applicable
16. Sample Size		
16a. How sample size was determined	How sample size was determined, including all assumptions supporting the sample size calculation.	Paragraphs 6
16b. Interim analyses and stopping criteria	Explanation of any interim analyses and stopping guidelines.	Not applicable
17. Randomisation		
17a. Sequence Generation	Who generated the random allocation sequence and the method used.	Paragraphs 5
17b. Type of Randomisation	Type of randomisation and details of any restriction (eg, stratification, blocking, and block size).	Paragraphs 5
18. Allocation concealment mechanism	Mechanism used to implement the random allocation sequence (eg, central computer/telephone; sequentially numbered, opaque, sealed containers), describing any steps to	Paragraphs 5

	conceal the sequence until interventions were assigned.	
19. Implementation	Whether the personnel who enrolled and those who assigned participants to the interventions had access to the random allocation sequence.	Paragraphs 5
20. Blinding		
20a. Who was blinded	Who was blinded after assignment to interventions (eg, participants, care providers, outcome assessors, data analysts).	Paragraphs 11
20b. How blinding was achieved	If blinded, how blinding was achieved and description of the similarity of interventions.	Paragraphs 11
21. Statistical methods		
21a. Comparing groups	Statistical methods used to compare groups for primary and secondary outcomes, including harms.	Paragraphs 12
21b. Definition of who is included in each analysis	Definition of who is included in each analysis (e.g., all randomised participants), and in which group.	Paragraphs 6
21c. Missing Data	How missing data were handled in the analysis.	Paragraphs 12
21d. Additional Analyses	Methods for any additional analyses (eg, subgroup and sensitivity analyses), distinguishing pre-specified from post hoc.	Not applicable
22. Participant flow, including flow diagram		Paragraphs 12
22a. Participant Numbers	For each group, the numbers of participants who were randomly assigned, received intended intervention, and were analysed for the primary outcome.	Table 1
22b. Losses and exclusions	For each group, losses and exclusions after randomisation, together with reasons.	Table 1
23. Recruitment		
23a. Dates	Dates defining the periods of recruitment and follow-up for outcomes of benefits and harms.	Ethics and Informed Consent
23b. Reasons for stopping recruitment	If relevant, why the trial ended or was stopped.	Not applicable
24. Intervention and comparator delivery		
24a. As Administered	Intervention and comparator as they were actually administered (eg, where appropriate, who delivered the intervention/comparator, whether participants	Not applicable

	adhered, whether they were delivered as intended (fidelity)).	
24b. Concomitant Care	Concomitant care received during the trial for each group.	Not applicable
25. Baseline Data	A table showing baseline demographic and clinical characteristics for each group.	Table 1 & 2
26. Numbers analysed, outcomes, and estimation	For each primary and secondary outcome, by group:<br>• the number of participants included in the analysis.<br>• the number of participants with available data at the outcome time point.<br>• result for each group, and the estimated effect size and its precision (such as 95% confidence interval).<br>• for binary outcomes, presentation of both absolute and relative effect size.	Paragraphs 13-16
27. Harms	All harms or unintended events in each group.	Not applicable
28. Ancillary Analyses	Any other analyses performed, including subgroup and sensitivity analyses, distinguishing pre-specified from post hoc.	Not applicable
**Discussion**		
29. Interpretation	Interpretation consistent with results, balancing benefits and harms, and considering other relevant evidence.	Paragraphs 17-20
30. Limitations	Trial limitations, addressing sources of potential bias, imprecision, generalisability, and, if relevant, multiplicity of analyses.	Paragraphs 22
